# Transnasal endoscopic resection of nasal chondromesenchymal hamartoma in infancy: an analysis of 5 cases

**DOI:** 10.1186/s12887-021-03082-4

**Published:** 2022-01-06

**Authors:** Zheng Jie Zhu, Qi Huang, Lan Cheng, Jun Yang

**Affiliations:** Department of Otolaryngology Head and Neck Surgery, Xinhua Hospital, Shanghai Jiaotong University, School of Medicine, Shanghai, 1665 Kongjiang Road, Shanghai, 200092 China

**Keywords:** Nasal chondromesenchymal hamartoma, Endoscopy, Infancy

## Abstract

**Background:**

Nasal chondromesenchymal hamartomas (NCMHs) are extremely rare benign tumors that most commonly affect children in the first year of life. The purpose of this study was to investigate and summarize the characteristics of NCMH cases and the efficacy of transnasal endoscopic resection of NCMHs.

**Methods:**

This is a retrospective study including 5 cases of infant diagnosed as NCMH between April 2016 and April 2020. Diagnostic techniques include nasoendoscopy, computerized tomography (CT) scan, magnetic resonance imaging (MRI) with contrast and microscopic and immunohistologic studies. Data collected included patient demographics, patient symptoms, radiographic findings, characteristics of tumor growth, follow-up time, recurrence, and postoperative complications.

**Results:**

In 5 cases, 3 were males and 2 were females who aged 1, 2, 3, 6 months and 1 year, respectively. The size of the mass measured 1.6 cm*1.9 cm*1.8 cm at its smallest and largest was 4.0 cm*3.5 cm*3.0 cm. All five patients underwent tumor resection via transnasal endoscopic approach. Four tumors were completely removed, and one underwent partial resection, which was completely resected by midfacial degloving operation 13 months after the first surgery. There was no postoperative complication. The current postoperative follow-up period was 1 to 4 years, and no recurrence has been observed.

**Conclusions:**

Complete surgical resection of NCHM is necessary to resolve the symptoms and prevent recurrence. Transnasal endoscopic approach is a safe and effective choice for pediatric NCMH patients.

## Background

NCMHs are over growth of mixed morphological structure composed of predominantly of mesenchymal and cartilaginous tissues. NCMHs are extremely rare benign tumors in clinical practice [1]. To date, as far as we know, fifty cases have been reported in the English literature [2]. NCMH has also been called “mesenchymoma”, “chondroid hamartoma”, “nasal hamartoma” and other names in the literature. The term: “Nasal chondromesenchymal hamartoma” was first suggested by McDermott et al. in 1998 [3] and has been used to date. NCHMs are mostly seen within the first 6 months of life [4]. The presentation and symptoms depend on the location and size of the tumor as well as involvement of surrounding structures. The appearance and radiologic findings of NCMH mimicking other congenital nasal neoplasm including meningocephalocele, embryonal rhabdomyosarcoma, teratoma, glioma and etc., thus NCMH is easily to be misdiagnosed clinically. A correct diagnosis is made from pathological findings.

In this report, we described our experience with 5 pediatric patients who presented to our department with NCMHs and underwent transnasal endoscopic surgery between April 2016 and April 2020.

## Methods

### Patients

We retrospectively reviewed all cases of NCMH submitted for surgical treatment between April 2016 and April 2020. During these period 5 cases of pediatric patients diagnosed as NCMH confirmed by pathological findings were treated in our department, among which 3 were males and 2 were females who aged 1, 2, 3, 6 months and 1 year, respectively. Data collected included patient demographics, patient symptoms, radiographic findings, characteristics of tumor growth, follow-up time, recurrence, and complications. Permission was obtained from the parents of the child to publish the information, including pictures.

### Diagonostic techniques

Nasoendoscopy was performed preoperatively in all 5 patients. After surface anesthesia with 0.01% epinephrine cotton piece with tetracaine, rigid nasal endoscopy was performed on both sides of the nasal cavity with a 2.7-mm Storz 0° endoscope.

All 5 patients underwent high-resolution CT scan and MRI with contrast. CT scans were performed with a SIEMENS Somatom Definition 64 slice dual source CT scanner. Technical parameters: continuous scanning in horizontal position, with a layer thickness of 1.00 mm and a soft tissue window field of view larger than 200 mm, about 90 ~ 110 levels. Scans ranged from superior to the frontal sinus, inferior to the lower edge of the earlobes, anterior to the nasal tip, and were reconstructed in parallel sagittal and coronal planes with a reconstructed slice thickness of 1.00 mm. MRI scans were performed with a GE Twin speed plus 1. 5 T and 3. 0 t GE HD × MRI scanner of head with 8-receiving channels phased-array coil. The high-resolution scanning sequence was a three-dimensional fast imaging employing steady state acquisition (FIESTA) high-resolution MRI scan with a slice thickness of 0.8 mm.

Microscopic and immunohistologic studies can provide definitive confirmation of a NCMH. Microscopically, NCMH is characterized by irregular islands of mature and immature hyaline cartilage with occasional binucleated chondrocytes. The islands of cartilage are well demarcated from the surrounding stromal tissues, which have a myxoid background [5]. Stromal and cartilage cells were positive for vimentin. S-100 immunoreactivity was evident in the lobules of mature cartilage and in scattered stromal cells. No cells were positive for cytokeratins and EMA.

## Results

In 5 pediatric patients, NCMHs were located in the left nasal cavity in 3 cases and in the right nasal cavity in 2 cases. All five patients were found to have unilateral nasal congestion soon after birth, accompanied with choking milk and snoring. In one of these patients, the tumor showed progressive growth over a period of 2 months, and in the other 4 cases, the tumors did not significantly increase in size during the course of disease. Data collected was shown in Table 1.

**Fig. 1:**
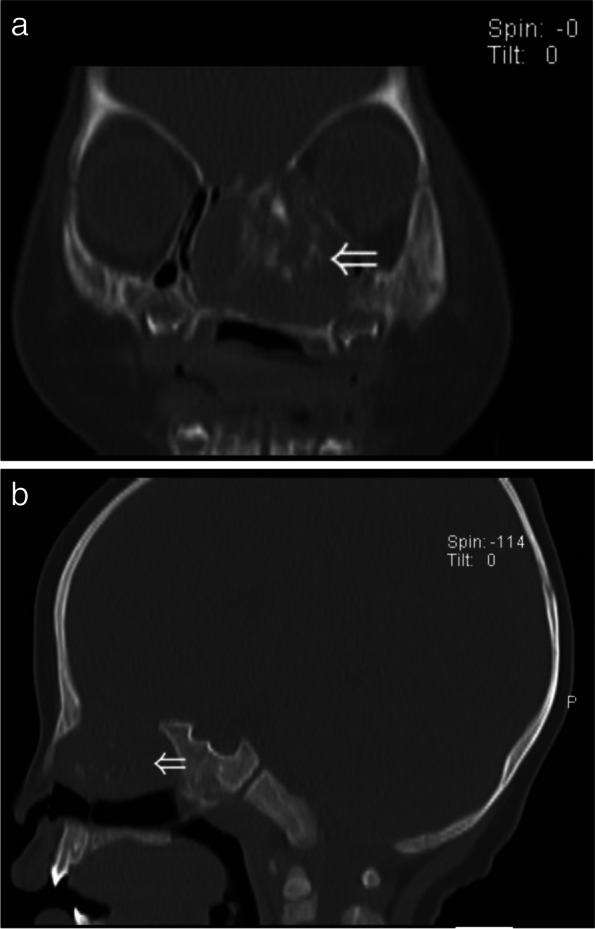
CT images. Coronal (**a**) sagittal (**b**) plane non-contrast images demonstrate a large soft-tissue density erosive mass occupying the left nasal cavity with extension into the left paranasal sinuses and occlusion of the nasopharnx. There is bony destruction of the anterior skull base and the left orbital wall along with multiple foci of calcification seen within the mass

**Table 1:** Case data of 5 children with NCMHs (in order of admission to hospital)

Case	Symptoms	Endoscopic findings	Radiologic findings	Intraoperative management	Postoperative follow-up
1	Nasal congestion after birth,epistaxis,choking milk and snoring	a pink, smooth, non-pulsatile polypoid mass filled the left nasal cavity	CT imaging revealed a soft-tissue density erosive mass measuring 3.2 × 4.1 cm2 in the left nasal cavity with local extension into the left paranasal sinuses and nasopharyngeal cavity with bony destruction of the anterior skull base and the left orbital wall (Fig. 1). MRI showed soft tissue mass with T1-weighted hypointensity and T2-weighted hyperintense, with multiple cyst-like signals inside. Post contrast T1-weighted image demonstrates heterogeneous enhancement (Fig. 2a, b). The surrounding tissue was obviously compressed, and the nasal septum was deviated to the right.	Subtotal resection of the tumor due to the young age of the infant	Further re-excision with midfacial degloving after 13 months, 4 years follow-up without recurrence
2	Nasal congestion, choking milk and feeding difficulty	a greyish-white,smooth mass occuping the left nasal cavity	Heterogeneous mass occupying the left nasal cavity, paranasal sinus and nasopharyx	Tumor total resection	4 years follow-up without recurrence
3	Nasal congestion and feeding difficulty	a greyish-white,smooth mass occuping the left nasal cavity	Soft-tissue density mass with calcification occupying the left nasal cavity, paranasal sinus and nasopharyx, with bony destruction of left orbital wall	Tumor total resection	3 years follow-up without recurrence
4	nasal congestion, mouth breathing, sleep snoring, episodes of breathing cessation during sleep shortly after birth, right epistaxis	a pink neoplasm occupying the right nasal cavity with significant left deviation of the nasal septum (Fig. 3a), ipsilateral choanal stenosis	CT imaging showed heterogeneous mass within the right ethmoid sinus, bone defects in the skull base, remodeling and expansion of the adjacent bony structures were seen which include ethmoid bony expansive destruction, partial absorption of the bone of the right orbital wall (Fig. 4). MRI revealed right nasal well-capsulated mass with T1-weighted hypointense, well-defined T2-weighted hyperintense myxoid and cystic components (Fig. 5). Post contrast T1-weighted image demonstrates heterogeneous enhancement	Tumor total resection without management of ipsilateral choanal stenosis because the contralateral choana was normal and unobstructed (Fig. 3b, Fig. 3c).	2 years follow-up without recurrence
5	Nasal congestion and snoring	a pink, solid appearing mass with suface overlying normal nasal mucosa	Tumor occupying the right nasal cavity and ethmoid sinus with bony destruction of skull base.	Tumor total resection	1 year follow-up without recurrence

At nasoendoscopy, a unilateral nasal mass with a smooth surface, grayish or pink in color, and soft or ductile texture was noted. Because of the infants’ nasal cavity were small and tumors were relatively large, all of the nasal septum showed various degrees of compressive deviation. The tumors were originated from the top of the nasal cavity, and the root was not palpable. One patient was accompanied with ipsilateral choanal stenosis. In one case, the tumor slightly enlarged when crying. One case with a huge mass in the nasal cavity and the ipsilateral choana could been seen, and there was no obstruction of the choana at all in the other two patients. Routine examination was performed in the nasal cavity without tumor, and no other congenital malformation was found in 5 cases.

Preoperative imaging findings are displayed in Table 1. The size of the mass measured 1.6 cm*1.9 cm*1.8 cm at its smallest and largest was 4.0 cm*3.5 cm*3.0 cm. CT examinations revealed soft-tissue density mass occupying the nasal cavity, which involved the ethmoid sinus and nasopharynx in 3 cases and the ethmoid sinus in 2 cases. Bony defect in the skull base was found in three patients and bony destruction of the ipsilateral obital wall in two patients. Of the 5 patients, 3 patients demonstrated multiple foci of calcification and with bony remodeling, and 2 demonstrated mutiple cystic components. MRI findings revealed that the masses were hypointense at T1-weighted images and hyperintense in T2-weighted images, and all 5 cases showed heterogeneous enhancement post-contrast. In 2 cases, multiple cystic components were seen which were consistent with CT imaging findings. The demarcation between the mass and adjacent structures was clear.

All five patients underwent tumor resection via transnasal endoscopic approach by using a 2.7-mm Storz 0° endoscope under general anesthesia. First, small pieces of tumor tissue were taken for biopsy via frozen section in order to quickly clarify the property of the tumor. Then, the tumors were suctioned to the root by using an microdebrider. Four tumors were completely removed, and one underwent partial resection, which was completely resected by midfacial degloving operation 13 months after the first surgery. In all 5 cases, the NCMHs originated from cribriform plate and the bone of cribriform plate was thin. In two cases, skull base defects were observed but without obvious cerebrospinal fluid leakage. One patient with ipsilateral choanal stenosis did not undergo extended surgery to correct the choanal atresia because the contralateral choana was normal and unobstructed. Intraoperative findings revealed that the NCMHs were capsulated, hypovascular, and may have cystic fluid or bone-like structures inside. Postoperatively, A gelatin sponge impregnated with antibiotics (such as neomycinsulphate, aureomycin, and polymyxin) was packed in the nasal cavity for hemostasis and prophylactic antibiotics were administered to prevent infection. The final pathological report all confirmed the diagnosis of NCMH (Fig. 6).

**Fig. 2:**
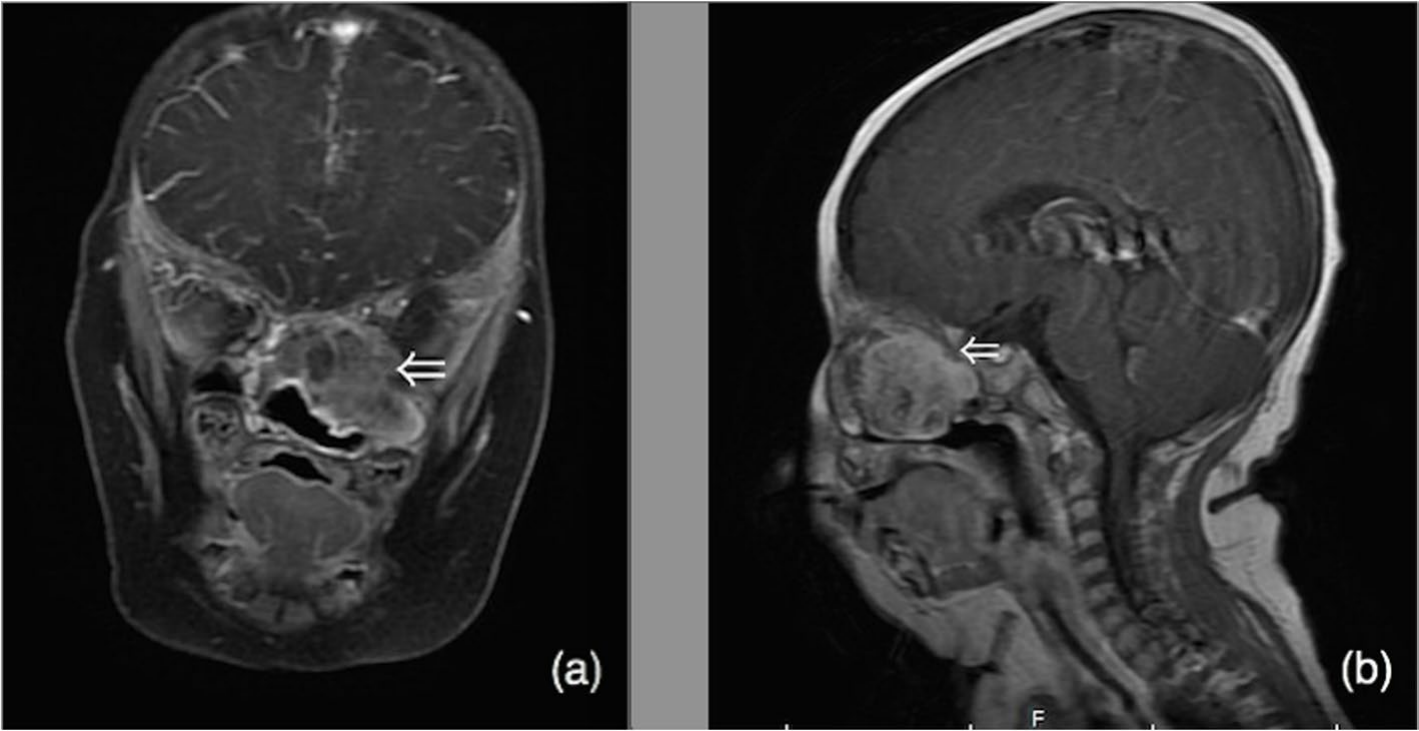
MRI image (**a**) Coronal post contrast T1-weighted image demonstrates left nasal mass with multiple cyst-like signals and heterogeneous enhancement. (**b**) Sagittal post contrast T1-weighted image showed a left nasal tumor with heterogeneous enhancement and a gap remained for ventilation in meatus nasi inferior

It was worth mention that in case 1, the neoplasm was initially considered the possibility of malignant germ cell tumor according to the preoperative imaging findings. In consideration of the young age of the infant, the first operation was performed via transnasal approach with the aim of clarifying diagnosis and relieving the symptoms. Intraoperatively, the tumor had the macroscopic appearance of red, tough nodule, and originated from the cribriform plate, and filling the entire left nasal cavity. Frozen-section histology reported as a low-grade malignancy of mesenchymal tissue, final result awaiting paraffin reporting. Intraoperatively, the mass was suctioned as much as possible to expose the ipsilateral choana by using an microdebrider in order to improve nasal ventilation and relieve the compression of the obit wall by the tumor. Postoperatively, symptoms of nasal congestion and sleep disturbance were resolved meanwhile the feeding was improved, and weight was increased. 6 months after the first surgery, the symptom of nasal congestion was not obvious and feeding was good. MRI scan was regular done and the image showed a mass filled with the left nasal cavity, which was nearly the same as the first MRI but there was still a gap remained for ventilation in meatus nasi inferior (Fig. 2b). Since the symptoms were not obvious and the margins of the tumor were not clear, our Multi-disciplinary Team suggested a midfacial degloving operation for totally resection of the tumor in order to prevent recurrence after one-year-old. 13 months after the first operation, the symptoms of nasal congestion and sleeping snoring were reoccurred and preoperative MRI showed that the tumor filled the entire left nasal cavity. Thus, the tumor was totally resected by midfacial degloving operation successfully.

**Fig. 3:**
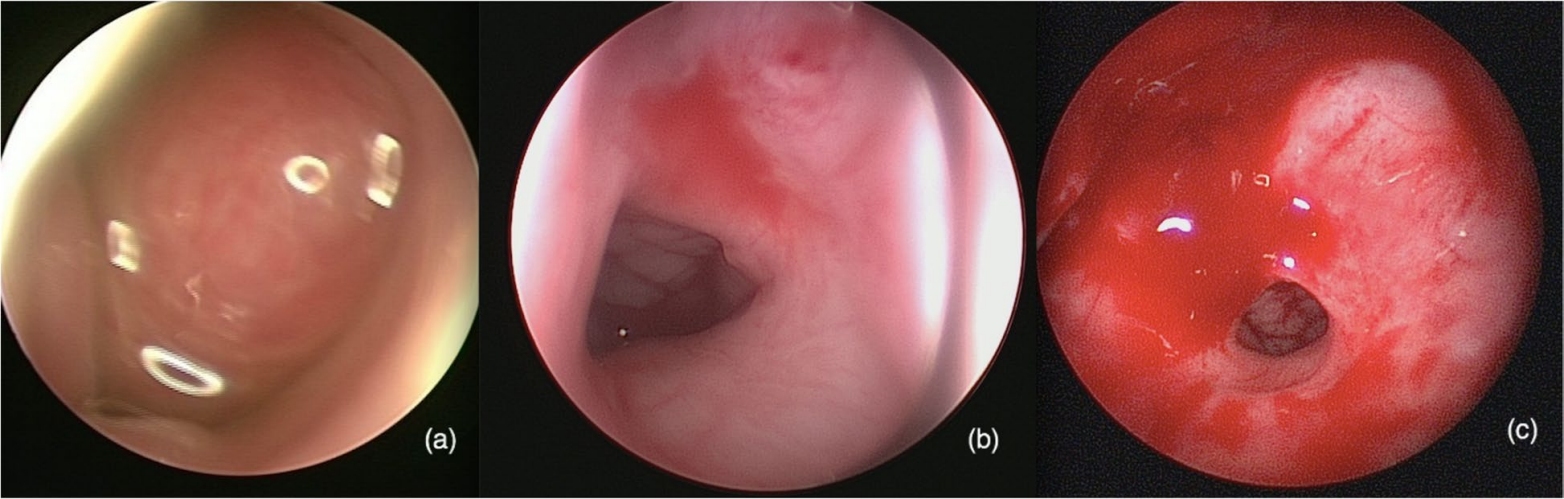
Nasoendoscopic examination (**a**) revealed a pink neoplasm occupying the right nasal cavity with significant left deviation of the nasal septum. Endoscpopic image (**b**) and (**c**) demonstrated patient with ipsilateral choanal stenosis and normal contralateral choana

There were no postoperative complications including postoperative hemorrhage, nerve impairments, vision disorder, cerebrospinal fluid leakage and etc. in any of the 5 patients. Regular outpatient follow-up was performed postoperatively, and the surgical cavity was cleared by nasoendoscopy 2 weeks postoperatively. Due to the young age of the affected infants, clearing is mainly aimed at removing incompletely degraded sponge, blood scabs and secretions in the nasal cavity. The clearing procedure should be gentle, less damaging and fast. Monthly nasal endoscopic follow-up examination was recommended for 3 month, and once 6 month thereafter. MRI examination was recommended at half year postoperatively and once 1 year thereafter. The current postoperative follow-up period is 1 to 4 years, and no recurrence has been observed. Specific results are shown in Table 1.

**Fig. 4:**
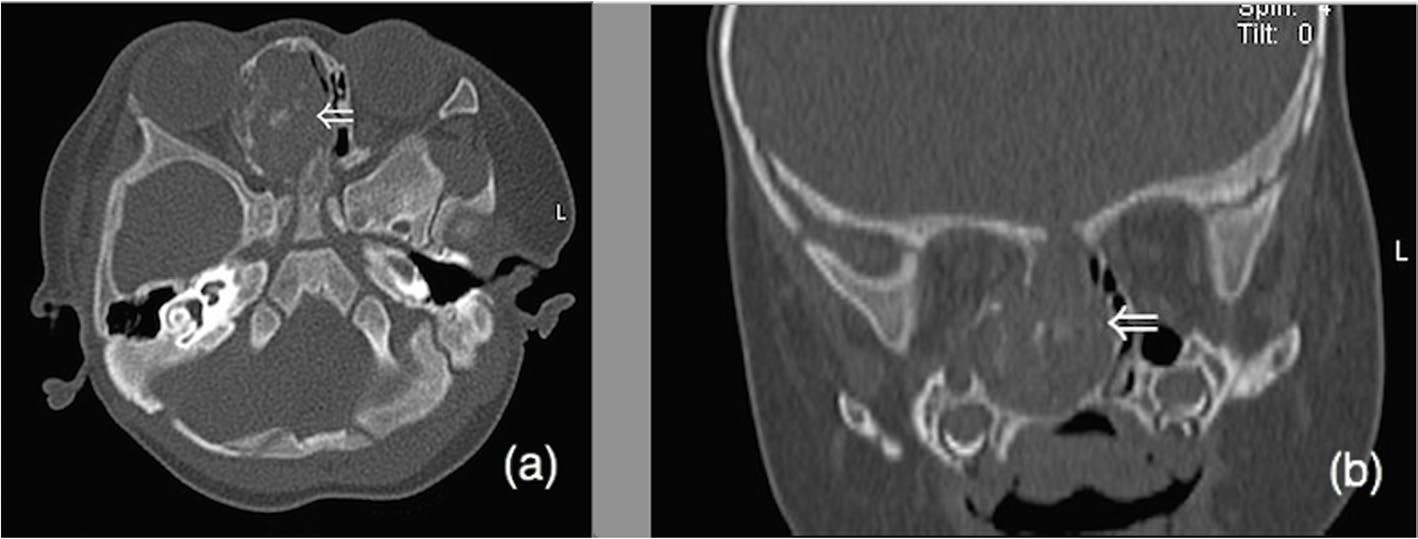
Axial (**a**) coronal (**b**) plane non-contrast images demonstrate a huge solid right intranasal mass with remodeling and expansion of the adjacent bony structures: ethmoid bony expansive destruction, partial absorption of the bone of the right orbital wall, deviation of the nasal septum and bone defects in the skull base

**Fig. 5:**
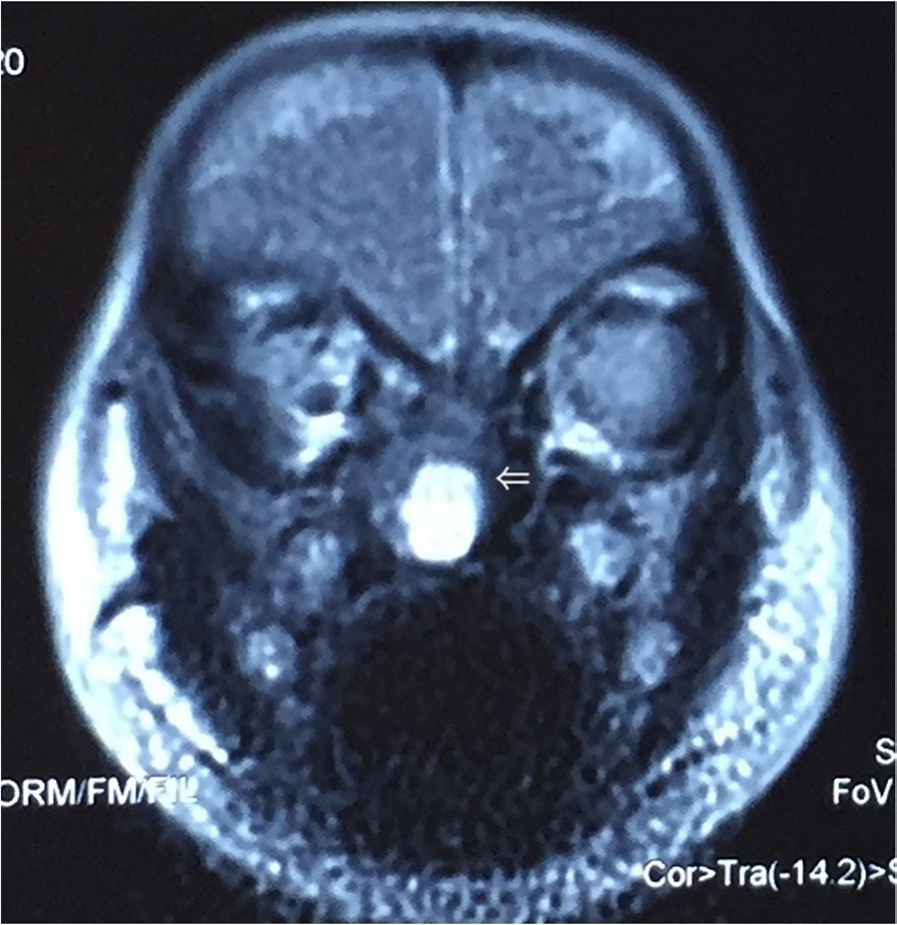
MRI Coronal image revealed right well-capsulated intranasal mass with T2-weighted hyperintense myxoid and cystic components

**Fig. 6:**
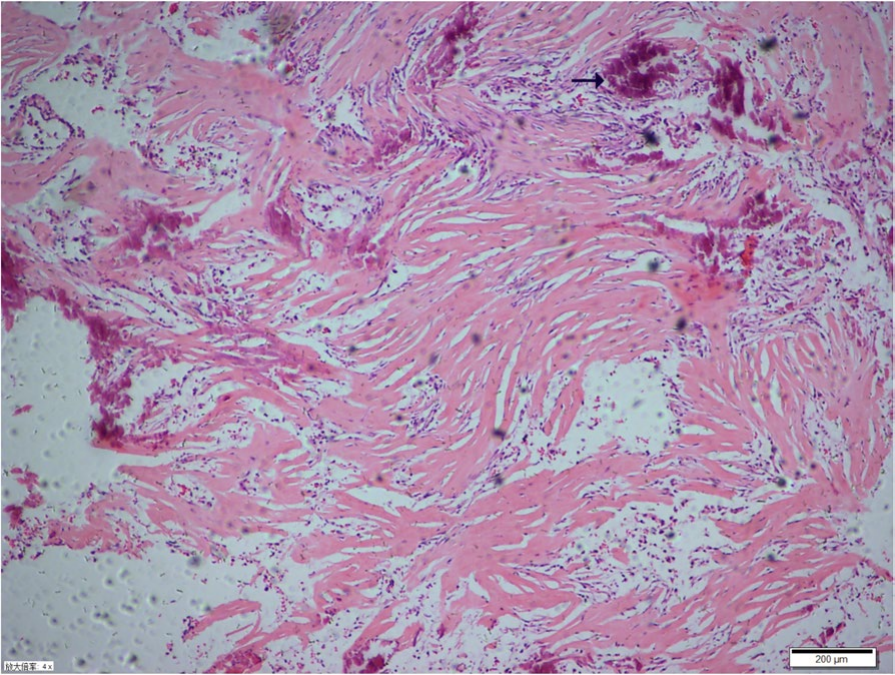
Photomicrograph demonstrates the components of the tumor with spindle cells, glial fibers and islands of cartilaginous calcification (arrow)

## Discussion

NCMH is an uncommon chondro-stromal proliferation of the nasal and sinus cavities, which is composed of one or more tissue components with mature diffentiation and disordered structure, showing circumscribed growth and no tendency of malignant degeneration. NCMH is an exceedingly rare tumor and mostly presents as an intranasal mass in infants in the first few weeks of life [4]. Neonatal nasal masses are rare, the overall incidence of these disorders has been reported to be around 1:20,000–1:40,000 live births [6]. In the English literature to date, there are approximately 52 reported cases of NCMH [1, 2]. Most cases presented in males; 38 male and 14 female, with a male to female ratio of 2.7:1 ratio. The mean age was 10.6 years (range:1 day-70 years). A large proportion of these patients were aged under 1 year at presentation (*n* = 20) and only 9 adult patients have been reported.

The exact etiology of NCMH is unknown. Most documented cases with characteristic features occurred in infants, thus NCMH was considered to be a congenital lesion. However, there are documented cases of adolescence with an asymptomatic childhood and even adults, with the oldest patient being 70 years old [7]. Thus, the hypothesis has been proposed that the tumor is caused by an underlying genetic predisposition in combination with specific stimulation that may include environmental or possibly hormonal in nature [5]. It has been also postulates that even when occurring in adolescence, lesions to have been present at birth in older children, the adolescent growth spurt causing an increase in size and subsequent symptoms [3]. A recent report highlighted the somatic DICER1 mutation rather than an inborn germline mutation, causing a harmatoma to form later in age [8].

The clinical manifestations of NCMH depend on the size and location of the lesion, which include respiratory and feeding difficulties, nasal congestion, rhinorrhea, epistaxis, hydrocephalus, visual disturbances, abnormalities of ocular motility, otitis media, recurrent sinusitis [9], usually presenting at a young age, mostly as neonates, infants and children. In our report, 5 cases of children manifested as obvious nasal congestion soon after birth, which leading to sleep snoring, episodes of breathing cessation and feed difficulties.

In terms of radiologic diagnosis, CT and MRI are useful to evaluate the size and the location of the mass as well as the status of adjacent structures, which include paranasal sinuses, orbit, and intracranial cavity. CT better visualizes osseous erosion and remodeling, MRI provides superior tissue characterization and delineation of extension into the adjacent structures. NCMHs can be locally aggressive with an expansive, destructive radiographic appearance, including displace of adjacent structure, bony destruction of cranium and/or the orbital cavity, which mimic malignancy. NCMHs appear as a non-encapsulated lesion with calcification on CT image, and bone erosion destruction and displacement of adjacent structures can be seen. MRI features include isointense on T1-weighted and slightly hyperintense on T2-weighted image, often with cystic components, heterogeneously enhancing post-contrast. The initial preoperative radiographic diagnosis of NCMH can be difficult and error-prone. A correct diagnosis is important to avoid potentially harmful therapies [10, 11]. Radiologic differential diagnosis should include meningoencephalocele, glioma, hemangioma, and embryonal rhabdomyosarcoma. Meningoencephalocele appears as hypo- or isodense anomaly on CT image, bony defects of skull base can often be seen. Abnormal signal mass involving the paranasal sinuses was connected with brain tissue on MRI. Glioma shows an expansive lesion comparable to the density of brain tissue on CT image, without bony destruction of skull base. Gliomas are hyperintense lesion on both T1-weighted and T2-weighted of MRI and post contrast T1-weighted image demonstrates poorly enhancement. Hemangiomas are hypointense on T1-weighted and hyperintense on T2-weighted on MRI, and markedly enhance post contrast. Rhabdomyosarcomas’ radiological features include isodense lesion, expansive and infiltrative growth, and adjacent bone destruction with resorption. In our report, CT image revealed skull base defect in 3 cases and the skull base bone integrity was found in all 3 cases during operation. Preoperative CT can show bone defect and destruction, but for newborns and infancts, because of the thin bone of skull base and relatively low bone density, the CT image may provide false-positive image diagnosis of bone defect. Therefore, for infants with nasal mass, enhanced MRI examination is preferable in preoperative differential diagnosis due to its superior soft tissue enhancement, and it is safer without radiation.

The treatment strategy of NCMH is complete surgical excision, usually achieving good outcome [12]. If the mass is confined to the nasal cavity, it is often amenable to transnasal endoscopic approach and achieves complete resection. An incomplete primary excision poses risk of recurrence as well as the possibility of continued tumor growth. A complete excision however is not always technically feasible only via transnasal approach with refractory mass, the surgery may combined with other approach include midfacial degloving surgery, lateral rhinotomy, craniofacial approach, and etc. [1]. In addition, it is essential to have neurosurgical involvement in patients with intracranial extension [13]. Due to the narrow nasal cavity of infanct, the endoscope for transnasal endoscopic surgery should be selected with smaller diameter. In our report, we used a 2.7-mm Storz 0° endoscope during operation. Accurate operation can be carried out under direct observation, and operation field can be clearly displayed, which can reduce the damage to the surrounding tissue. The NCMHs of 4 pediatric patients originated from ethmoid roof, where the bone is fragile, thus, not only should we achieved a total resection of the tumor to reduce the recurrence, but also should we operated carefully to prevent the bone destruction of the ethmoid roof which might lead to cerebrospinal fluid leakage.

## Conclusion

In summary, preoperative evaluation of a suspected NCMH should include nasoendoscopy, enhanced MRI scan and CT scan to evaluate the size and the location of the mass as well as the status of adjacent structures. Definitive verification can be provided by microscopic and immunohistologic studies. If presence of a NCHM is established, complete surgical resection of the mass is necessary to resolve the symptoms and prevent recurrence and secondary vision developmental sequela. Due to the advantages of clear vision, less trauma and fewer complications, transnasal endoscopic approach is the first choice for pediatric NCMH patients.

## Data Availability

The datasets used and/or analysed during the current study available from the corresponding author on reasonable request.
